# Searching the Clinical Fitness Landscape

**DOI:** 10.1371/journal.pone.0049901

**Published:** 2012-11-14

**Authors:** Margaret J. Eppstein, Jeffrey D. Horbar, Jeffrey S. Buzas, Stuart A. Kauffman

**Affiliations:** 1 Department of Computer Science, University of Vermont, Burlington, Vermont, United States of America; 2 Department of Pediatrics, University of Vermont, Burlington, Vermont, United States of America; 3 Department of Mathematics & Statistics, University of Vermont, Burlington, Vermont, United States of America; 4 Vermont Oxford Network, Burlington, Vermont, United States of America; 5 Complex Systems Center, University of Vermont, Burlington, Vermont, United States of America; Institute for Systems Biology, United States of America

## Abstract

Widespread unexplained variations in clinical practices and patient outcomes suggest major opportunities for improving the quality and safety of medical care. However, there is little consensus regarding how to best identify and disseminate healthcare improvements and a dearth of theory to guide the debate. Many consider multicenter randomized controlled trials to be the gold standard of evidence-based medicine, although results are often inconclusive or may not be generally applicable due to differences in the contexts within which care is provided. Increasingly, others advocate the use “quality improvement collaboratives”, in which multi-institutional teams share information to identify potentially better practices that are subsequently evaluated in the local contexts of specific institutions, but there is concern that such collaborative learning approaches lack the statistical rigor of randomized trials. Using an agent-based model, we show how and why a collaborative learning approach almost invariably leads to greater improvements in expected patient outcomes than more traditional approaches in searching simulated clinical fitness landscapes. This is due to a combination of greater statistical power and more context-dependent evaluation of treatments, especially in complex terrains where some combinations of practices may interact in affecting outcomes. The results of our simulations are consistent with observed limitations of randomized controlled trials and provide important insights into probable reasons for effectiveness of quality improvement collaboratives in the complex socio-technical environments of healthcare institutions. Our approach illustrates how modeling the evolution of medical practice as search on a clinical fitness landscape can aid in identifying and understanding strategies for improving the quality and safety of medical care.

## Introduction

Wright [1] introduced the concept of visualizing biological evolution as search of a “fitness landscape”, where an individual's position in the landscape is determined by its N heritable characteristics (“features”) and the height of the landscape at any given location corresponds to the reproductive success (“fitness”) of the individual. The distance between individuals on the landscape corresponds to the dissimilarity in their features. Since selective processes tend to favor individuals with higher fitness, evolving populations will generally move uphill in the fitness landscape, subject to stochastic effects. Where different features interact nonlinearly to determine fitness, the landscape becomes rugged (fitness becomes less correlated in feature space) and may contain multiple peaks of varying elevations, making it more difficult to navigate upward on the terrain. In biological systems, it is widely recognized that nonlinear (epistatic) gene-gene and gene-environment interactions are ubiquitous [2]–[3], motivating the development of complex fitness landscape models [4]–[5]. The fitness landscape metaphor and various complex landscape models have subsequently been widely applied in the context of developing effective computational approaches to combinatorial optimization problems [6]–[8] as well as for modeling technological and organizational improvement [9]–[12]. Health care systems can also be viewed as complex adaptive systems of interacting components and processes [13]–[15], motivating Berwick [16] to suggest that, in many circumstances, learning by doing in small, local tests may be more effective than large-scale randomized clinical trials in achieving health care improvements.

We build off these ideas by applying a fitness landscape metaphor and model to the problem of searching for improvements in health care. We consider populations of health care institutions with variations in health care practices and interventions that result in differential patient survival rates at different institutions, and compare the effectiveness of two search approaches that are unique to the way health care institutions seek to make changes in their practices and interventions when seeking to improve patient outcomes.

Marked variations in clinical practices and patient outcomes among healthcare providers, which cannot be explained by differences in patient characteristics, were first reported over 40 years ago [17] and have since been confirmed for a wide range of medical and surgical conditions ([18]–[19], *The Dartmouth Atlas of Healthcare* <http://www.dartmouthatlas.org/>, *NHS Atlas of Variation in Healthcare 2011* http://www.sepho.org.uk/extras/maps/NHSatlas2011/atlas.html). This unexplained variation reflects the difficulty in reaching consensus on which combinations of practices are both safe and effective, and suggests that there are major opportunities for improving the quality and safety of medical care [20]. The gold standard for testing the safety and efficacy of clinical practices is the multi-institutional randomized controlled trial (RCT) in which the entire healthcare community can learn from the published results of determinate trials conducted at a subset of institutions. However, multi-institutional RCTs are rarely designed to discover potential interactions between the clinical practices being tested and the local contexts of different institutions [21], despite an increasing recognition that such interactions often exist [21]–[26]. Furthermore, it has been suggested that the findings of RCTs may often be accurate measures of the prevailing bias rather than the truth [27]. Despite these limitations, there is consensus that RCTs are essential for testing novel and experimental therapies. However, for achieving improvement in day-to-day practices in the complex terrain of healthcare institutions, Berwick refers to RCTs as “an impoverished way to learn” and suggests that learning from experience while doing has an important role to play in improving the quality of healthcare [28]. Indeed, many clinicians are now participating in organized quality improvement collaboratives (QICs), in which teams from different healthcare organizations exchange information on current practices and outcomes. QIC members identify potentially better practices in use by teammates and then try them out in the local context of their home institutions [28]–[31]. The current evidence supporting QICs is positive but limited, and the effects cannot be predicted with certainty [32]. In their systematic review of 1104 quality improvement articles, Schouten et al. [32] found only 9 studies that used a controlled approach for assessing the efficacy of QICs, of which 7 reported significant effects on some of the identified outcomes. Research in quality improvement continues to seek an understanding of which factors contribute to the effectiveness of QICs [33]–[38]. Some are concerned that QICs may lead to the adoption of ineffective or inferior practices because they lack the statistical rigor of RCTs [39]. Unfortunately, there is little theory to guide clinicians in assessing the relative merits of these two very different approaches to healthcare improvement, or in ascertaining the circumstances in which one strategy can be expected to yield better overall improvements in patient outcomes.

The aim of this paper is to explore the potential relative advantages and disadvantages of RCTs and QICs under various theoretical scenarios. We first frame the problem of health care improvement as search of a clinical fitness landscape. We then develop an agent-based model through which we examine the relative ability of agents, representing health care institutions, in navigating clinical fitness landscapes of varying complexity when using search strategies modeled after QICs or RCTs. The results of our simulations indicate that a search strategy modeled after QICs generally leads to greater improvements in health outcomes under a wide range of conditions than one modeled after RCTs, due to a combination of reduced sensitivity to sample size and an increased ability for agents to respond differently in different local contexts (i.e., in different regions of the same landscape). Interesting interactions are discovered between the ruggedness of the clinical fitness landscape, the feature selection strategy, and the initial similarity of agents.

## Methods

### Framing Health Care Improvement as a Search Problem

To study the relative efficacy of RCTs and QICs, we frame the problem as a combinatorial search of a high-dimensional clinical fitness landscape. Different healthcare institutions are modeled as agents that are cooperatively searching this landscape, ever trying to move to higher elevations. Since (i) RCTs and QICs are conducted, assessed, and acted on at the institutional or multi-institutional level, (ii) different subsets of health care institutions participate in different multi-institutional RCTs and QICs at different times, and (iii) each institution adopts its own culture and set of routine health care practices in seeking to improve health outcomes of their patients, an individual health care institution is an appropriate granularity of scale for a defining a search agent for the purposes of comparing RCT and QIC search strategies. Potential clinical practices, interventions, and other modifiable characteristics of individual healthcare organizations are modeled as dimensions in the landscape, collectively termed features or practices. When there are no interactions between different features, the clinical fitness landscape will be smooth, with a single peak, and relatively easy to navigate. However, as the frequency of feature interactions increases, landscapes become increasingly rugged and may contain multiple peaks [5]. We compare search on simulated clinical fitness landscapes of varying ruggedness and under varying conditions using strategies intended to capture the most salient distinguishing aspects of RCTs and QICs.

### Agent-Based Modeling

#### Clinical Fitness Landscape Model

We simulate clinical fitness landscapes as high dimensional logistic functions, in which combinations of feature values associated with different health care institutions specify different locations on the landscape, with associated elevations corresponding to the probability of beneficial patient outcomes at those institutions. Feature interaction terms in this model capture the notion that clinical practices and interventions may perform differently in different contexts, or in the presence of different co-interventions. Specifically, the probability of positive patient outcomes, hereafter synonymously referred to as survival probability or fitness, treated at a given healthcare institution (agent) is defined as follows: 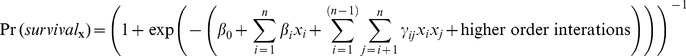
(1)where **x** comprises a vector of *n* binary features (*x_i_*∈{−1,1}) of an agent; the binary feature values represent presence or absence of the use of a specific practice, intervention, or other modifiable characteristic of the institution. This landscape model is similar to NK landscape models (4–5) in that it uses binary features and has tunable ruggedness. However, it is more suited to model the clinical fitness landscape because, unlike the NK model, (i) the logistic transformation returns values in the range 0 to 1, which can be interpreted as survival probabilities, (ii) the model allows complete flexibility in determining the number, strength, and direction of interactions of a specified order, and (iii) medical treatment effects and interactions are commonly analyzed using logistic regression models [40]. For the simulations reported here, we modeled landscapes with *β_0_* = 0. This can be interpreted as reflecting a shared history of prior learning in which consensus has already been reached on features not explicitly modeled, such that the mean survival probability of randomly placed agents will be 0.5 at the start of the simulations. We assumed main effects for all *n* features (non-zero coefficients *β_i_*), a specified number of 2-feature interactions (non-zero coefficients *γ_ij_*), and no higher order interactions. For a given landscape, specific values for included coefficients *β_i_* and *γ_ij_* were randomly drawn from a normal distribution with a mean of 0 and a standard deviation of *L* ^−0.5^, where *L* is the total number of terms in the model. This choice of standard deviation ensures a unimodal distribution of possible survival probabilities, with relatively few values near 0 or 1. In a given instantiation of a landscape, the strength of the effect of individual treatments and interactions can vary widely, depending on the particular realized values of the random coefficients. We note that one could alternatively sample from longer-tailed distributions to allow for a few treatments or interactions with even larger effects. The observed survival rate of *N_p_* patients treated by the agent is defined as the average of *N_p_* Bernoulli trials with survival probability as determined by Eq. (1), where the stochasticity in the Bernoulli trials represents heterogeneity in patient-level responses.

#### RCT Search Strategy

The RCT strategy we model corresponds to a world in which large scale observational studies are used to identify potentially effective practices already in use at some institutions before subjecting them to rigorous testing in multi-center pragmatic RCTs, and features found to be significantly better (p<0.05) are rapidly adopted by all other institutions. Specifically, during each trial step of the RCT strategy for the experiments reported here, one group of 10 randomly selected agents participates in a multi-center trial to test one feature, selected as the feature that exhibits the greatest difference in mean feature value between all agents above and below the population-wide median survival probability (a.k.a. global feature selection). Half of the patients from each of the participating 10 agents are tested with the selected feature value set to −1, and half with the feature value set to 1 (i.e., each RCT trial enrolls 10×*N_p_* patients). A two-sided significance test is then performed on the observed fitness of the pooled results for each treatment group; if the results are significant at the 5% significance level, all 100 agents in the population adopt the better feature value. Features found to be determinate in RCT trials are never retested.

#### QIC search strategy

In contrast, the QIC strategy we model corresponds to a world in which information on practices and outcomes is shared among teams from multiple institutions. Team members use this information to individually determine a promising practice to evaluate and individual institutions make decisions to adopt new practices based solely on the observed results of trying them in their local contexts, without regard to statistical significance. Specifically, during each trial step of the QIC strategy of the experiments reported here, 100 agents are randomly partitioned into 10 teams of 10. Each agent (except the fittest agent in each team) independently selects one feature with the greatest difference in mean feature value between teammates with survival probability above and below the survival probability of the agent, and such that the consensus feature value of the fitter agents is different than the current feature value for the agent (a.k.a. local feature selection). These agents then conduct single-center trials (*N_p_* patients each) on the selected feature in their local contexts, where half of the agent's cases are tested with the selected feature value set to −1, and half tested with the feature value set to 1. QIC agents adopt the feature observed to yield higher survival, without regard to statistical significance. Although real-world QICs often differ in the particular search strategies they employ, the strategy modeled here incorporates the key concepts of multi-organizational QICs [32], [41], [42].

#### Simulations

**Figure 1:**
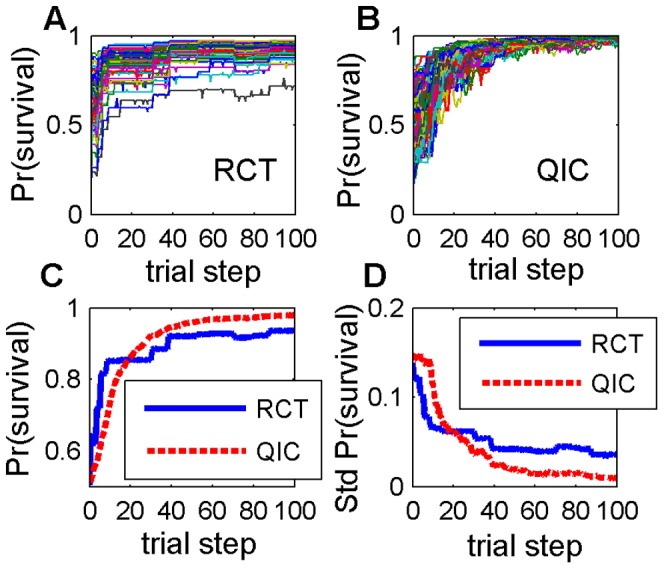
Representative simulation. One simulation with 495 two-feature interaction terms, a clustered initial distribution, and *N_p_* = 40. (A) Expected survival probabilities of each of 100 agents using RCT search; (B) expected survival probabilities of each of 100 agents using QIC search; (B) expected survival probabilities averaged over the 100 agents, (D) standard deviation of expected survival probabilities of the 100 agents. RCT refers to a search strategy modeled after Randomized Controlled Trials; QIC refers to a search strategy modeled after Quality Improvement Collaboratives.

We assessed the effectiveness of the two search strategies under a variety of conditions by varying 3 factors, as follows. (I) The patient sample size per agent in each trial step was varied as *N_p_* ∈{40, 80, 160, 320}. (II) The ruggedness of the fitness landscape was varied by changing the number of feature interactions; we generated 150 random 100-feature landscapes, fifty each with a random 0, 495 (10%), or 2475 (50%) of the 4950 possible 2-way interaction terms, representing 3 levels of increasing landscape ruggedness. (III) The initial locations of the 100 RCT or 100 QIC agents were either uniformly scattered or randomly clustered on the landscape, representing the degree of similarity of local contexts of agents at the start of the search; specifically, the median feature dissimilarity (normalized Hamming distance) between all pairs of agents was initially either 0.5 (scattered) or 0.1 (clustered). Both of these initialization strategies assume no prior knowledge of the relationship between health outcomes and the treatments for which consensus has not yet been achieved. Simulations were paired, in that separate populations of either 100 RCT or 100 QIC search agents were initially placed at random, but identical, sets of locations on an identical simulated clinical fitness landscape, and each population was then allowed to search the landscape for 100 trial steps. A representative paired simulation with *N_p_* = 40, 495 interaction terms, and a clustered initial distribution is shown in Figure 1.

## Results

**Figure 2:**
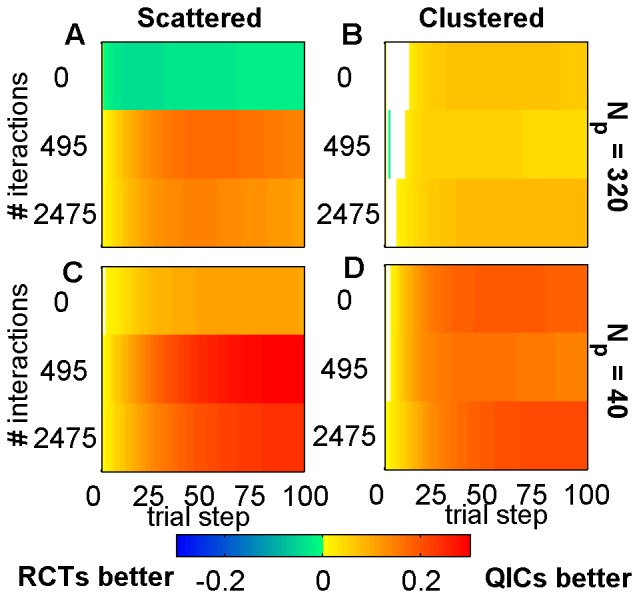
Differences in integrated survival probabilities. Survival probabilities using QIC search minus those using RCT search, integrated from the start of the simulation up through the indicated trial step and averaged over all 100 agents and all 50 random landscapes for scattered (A,C) or clustered (B,D) initial distribution and *N_p_* = 320 (A,B) or *N_p_* = 40 (C,D). Hot colors indicate where QIC search outperformed RCT search, and cool colors the opposite. All comparisons were significantly different at the p<0.01 level (2-sided paired t-test), except for regions shown in white. RCT refers to a search strategy modeled after Randomized Controlled Trials; QIC refers to a search strategy modeled after Quality Improvement Collaboratives.

We found that the QIC search strategy consistently resulted in significantly higher patient survival probabilities than the RCT search strategy under nearly all conditions tested (Fig. 2). Detailed comparisons under various conditions are described below.

### Searching from scattered initial agent distributions with large sample sizes

**Figure 3:**
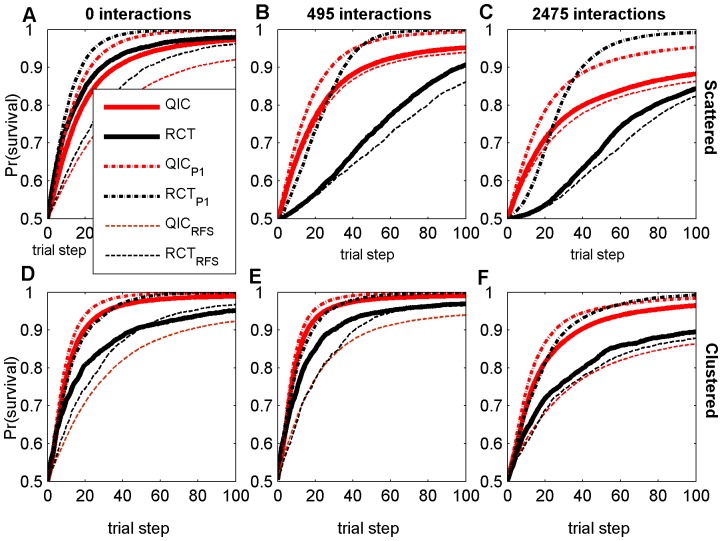
Survival probabilities at each trial step with *N_p_* = 320. Results are averaged over all 100 agents and all 50 random landscapes for each parameter combination, with either scattered (A–C) or clustered (D–F) initial distribution, and either 0 (A,D), 495 (B,E), or 2475 (C,F) random two-feature interaction terms. RCT refers to a search strategy modeled after Randomized Controlled Trials; QIC refers to a search strategy modeled after Quality Improvement Collaboratives. A subscript of P1 refers to simulations with no patient variation (100% power); a subscript of RFS refers to modified search strategies using Random Feature Selection.

**Figure 4:**
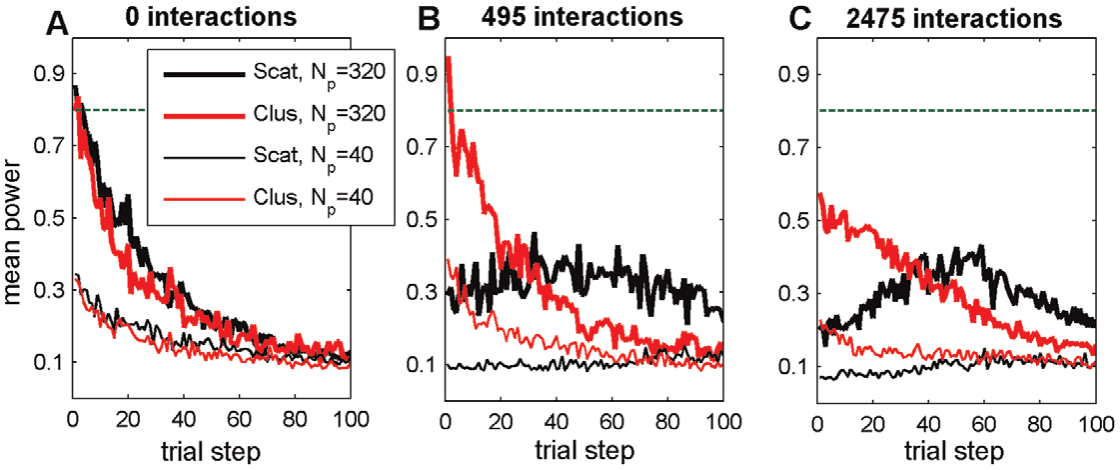
Actual power of RCT trial simulations. Results are averaged over 50 random landscapes with (a) 0, (b) 495, or (c) 2475 two-feature interaction terms. RCT refers to a search strategy modeled after Randomized Controlled Trials. Scat refers to a scattered initial distribution of agents, Clus refers to a clustered initial distribution of agents. *N_p_* is the number of patients per agent per trial step. The horizontal green line is the desired minimum power of 0.8.

The only circumstance tested in which RCT search resulted in higher patient survival was with large sample sizes on smooth landscapes and when agents were initially uniformly scattered. Even in this case, RCT search only slightly outperformed QIC search (Figure 3A, compare thick solid lines). In this relatively simple case, the rate of progress decreases monotonically for both search strategies due to the diminishing returns of improvement as fitness increases (Figure 3A). However, this decrease in the rate of progress is most pronounced in RCT search because, even on smooth landscapes with 3200 patients per trial, the power of RCT trials drops precipitously as fitness increases (Figure 4A). As the number of feature interactions increases, the power of initial RCT trials is dramatically reduced (compare Figure 4A,B,C, black lines), which is reflected by a sluggish initial rate of progress by RCT searchers (compare Figure 3A,B,C, thick black lines). As the RCT agents in an initially scattered population slowly become more similar following determinate trials, the average power of the RCT trials on rugged landscapes exhibits a transient increase, although still remaining well below the 0.8 target level of most real clinical trials (Figure 4, black lines). Because of this low power, and because determinate results may not be beneficial for agents with different local contexts when feature interactions are present, RCT search does relatively poorly on rugged landscapes with scattered initial distributions, even with large sample sizes (Figs. 3B,C, solid lines).

### Searching from clustered initial agent distributions

We performed baseline simulations using uniformly scattered initial agent distributions, because these provide unbiased initial starting points for the search, with maximal coverage of all regions in the search space. However, given that current healthcare institutions are part of a larger community that has already benefited from a long history of improvement and information-sharing, a clustered initial distribution of feature values in agents may be more realistic than a scattered initial distribution. It is therefore of interest that on smooth landscapes an initial clustering of agents actually hurt the performance of RCTs but helped that of QICs (compare thick solid lines on Figure 3A,D), while initial clustering improved the performance of both strategies on landscapes with feature interactions (compare thick solid lines on Figure 3B,C to Figure 3E,F). A clustered initial distribution improves the initial power of early RCT trials on rugged landscapes relative to a scattered initial distribution (Figure 4B,C, compare red lines to black lines), because trial participants have greater similarities in local contexts. However, a clustered initial distribution also improves QIC search, because agents can learn more rapidly from other agents with greater similarities in local contexts. In all cases tested, QICs outperformed RCTs when the initial distribution of agents was clustered (Figure 2B,D).

### Effect of Sample Size and Power

**Figure 5:**
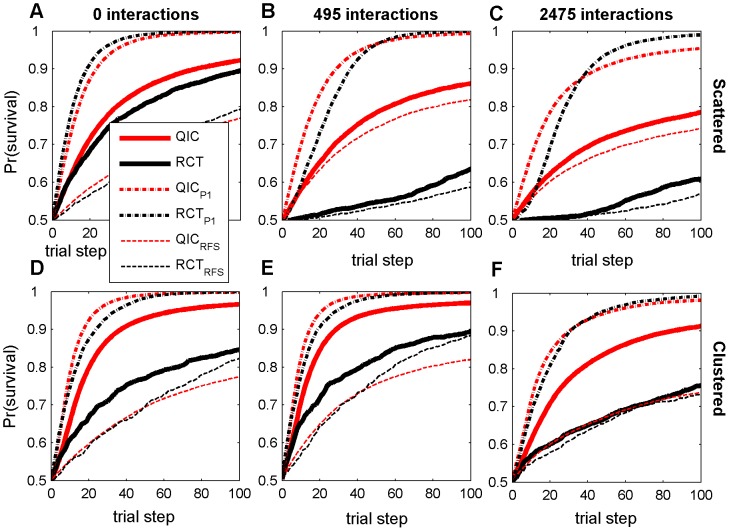
Survival probabilities at each trial step with *N_p_* = 40. Results are averaged over all 100 agents and all 50 random landscapes for each parameter combination, with either scattered (A–C) or clustered (D–F) initial distribution, and either 0 (A,D), 495 (B,E), or 2475 (C,F) random two-feature interaction terms. RCT refers to a search strategy modeled after Randomized Controlled Trials; QIC refers to a search strategy modeled after Quality Improvement Collaboratives. A subscript of P1 refers to simulations with no patient variation (100% power); a subscript of RFS refers to modified search strategies using Random Feature Selection.

As the sample size decreases, QIC search gains an increasingly greater relative advantage over RCT search under all levels of landscape ruggedness and initial distributions of agents. This is because the adopt-if-better approach of QIC searchers make them relatively less sensitive to sample size than RCT searchers, which require p<0.05 significance in trial results in order to make progress. For example, in the smallest sample sizes tested (*N_p_* = 40; i.e., 400 patients per 10-center RCT trial and 40 patients per single center QIC trial), QIC search consistently achieved significantly higher patient survival than RCT search (Figure 2C,D; Figure 5, thick solid lines). To further elucidate the role of power on performance, we ran additional simulations of the two search strategies with no simulated patient variation (shown in Figure 3, dash-dot lines labeled RCT_P1_ and QIC_P1_). In this case, where power is 100%, RCT_P1_ becomes competitive with QIC_P1_, even on rugged landscapes (Figure 4, dash-dot lines), indicating that low statistical power is a major limitation in RCT search of the rugged landscapes.

### Effect of Feature Selection Strategy

However, low statistical power is not the only reason that QIC search outperformed RCT search in simulations when agents are initially clustered. We also observed interesting interactions between the feature selection strategy, the amount of initial agent clustering, and the number of feature interactions. To explore the importance of the different feature selection strategies, we ran additional simulations in which both RCT and QIC search used random feature selection in determining which practices to test (shown in Figure 3, dashed lines labeled RCT_RFS_ and QIC_RFS_). This analysis shows that, on smooth landscapes, the global feature selection strategy of RCT search benefits from an initially scattered distribution of agents (note the large difference between RCT and RCT_RFS_ in Figure 3A). This occurs because dispersed agents sample more of the landscape and the global feature selection strategy is able to capitalize on this by selecting high impact features in early trials, thus facilitating rapid initial improvement. In contrast, when RCT search agents are clustered, improvements are rapid in early trials with global feature selection, but continued improvement is sustained longer with random feature selection (note how RCT_RFS_ ultimately outperforms RCT in Figure 3D). We attribute this to the fact that the random feature selection strategy facilitates discoveries in unexplored territory that the global feature selection strategy cannot reach. When feature interactions are present and the initial agent distribution is scattered (so local contexts vary widely), the local feature selection strategy of QIC search is not much better than random feature selection (compare QIC to QIC_RFS_ in Figure 3C,D), because there is little useful information an agent can learn from teammates that are located in very different parts of a rugged landscape. However, when the initial population is clustered, the local feature selection strategy is now much better than random feature selection (compare QIC to QIC_RFS_ in Figure 3E,F), because teammates are more likely to be climbing similar local topography in the clinical fitness landscape and therefore have much to learn from each other.

### Variability in Patient Outcomes and Agent Heterogeneity

**Figure 6:**
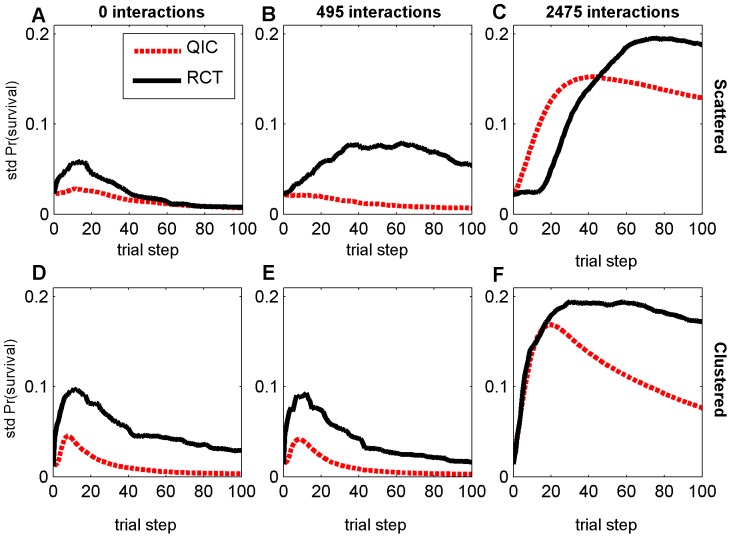
Standard deviation of average survival probabilities with *N_p_* = 320. Results are shown at each trial step, over 50 random landscapes for each parameter combination, with either scattered (A–C) or clustered (D–F) initial distribution, and either 0 (A,D), 495 (B,E), or 2475 (C,F) random two-feature interaction terms. RCT refers to a search strategy modeled after Randomized Controlled Trials; QIC refers to a search strategy modeled after Quality Improvement Collaboratives.

**Figure 7:**
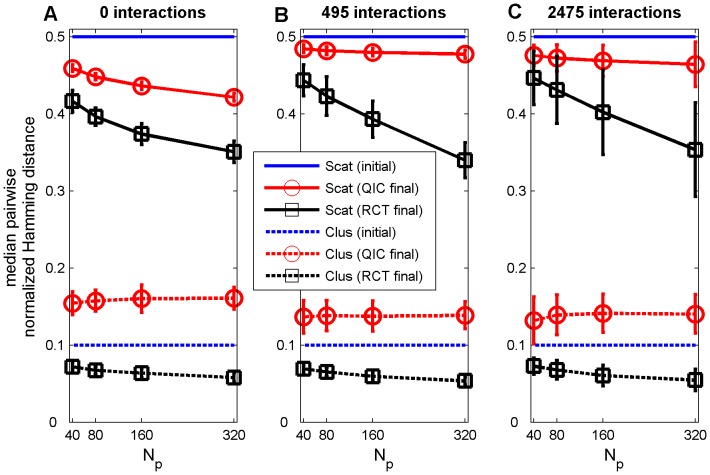
Median normalized Hamming distances. Degree of agent dissimilarity between all pairs of agents before (initial) and after (final) 100 trial steps for landscapes including (A) 0, (B) 495, or (D) 2475 two-feature interaction terms, averaged over 50 random landscapes for each parameter combination (error bars represent plus or minus one standard deviation), shown as a function of the number of patients *N_p_* treated by each agent during each trial step. RCT refers to a search strategy modeled after Randomized Controlled Trials; QIC refers to a search strategy modeled after Quality Improvement Collaboratives. Scat refers to a scattered initial distribution of agents, Clus refers to a clustered initial distribution of agents. *N_p_* is the number of patients per agent per trial step.

In addition to achieving higher overall average patient survival rates, QIC search also exhibits less variation in performance improvement, both between survival rates of individual agents on a given landscape (e.g., Figure 1d) and between survival rates of populations of agents across different random landscapes (Figure 6). Interestingly, this occurs despite the fact that RCT agents always share many more features in common than QIC agents at the end of 100 trial steps (Figure 7), due to the “what's good for some is good for all” approach to dissemination in RCT search in contrast to the “adopt if it's better for my institution” strategy of QIC search. For example, with large sample sizes and when starting from a clustered initial distribution, median feature dissimilarity between pairs of agents actually increased by about 50% in QIC search, while decreasing by about 50% in RCT search, as survival rates increased, reflecting the tendency of QIC searchers to adopt distinct combinations of practices that work well in their local contexts (Figure 7). These results highlight the fact that maintaining some variations in clinical practices may actually be beneficial for the quality and safety of healthcare, especially considering that some differing characteristics of healthcare institutions and the patients they serve are not modifiable and that consequently variations in local context will always exist to some degree. We note that any learning strategy in which agents make individual decisions will permit more heterogeneity than learning strategies that force agents to adopt consensus decisions, so qualitatively similar results are likely to be obtained under different assumptions for QIC and RCT search strategies.

## Discussion and Conclusions

Because the exact nature of the true clinical fitness landscape is unknown, we have examined the sensitivity of the QIC and RCT search strategies to some important model assumptions. We find that a search strategy modeled after QICs yields robust improvement in simulated patient outcomes regardless of the ruggedness of the clinical fitness landscape, degree of initial similarity in institutional practices, and patient sample size. In contrast, a search strategy with widespread dissemination of only those practices found to be statistically better in multicenter RCTs was very sensitive to these parameters, and only slightly out-performed QIC search in the (probably unrealistic) scenario comprising (i) smooth clinical fitness landscapes with no feature interactions, (ii) very large patient sample sizes, and (iii) hospital agents with initially uniformly random distributions of practices and interventions. If any of these three assumptions were relaxed, we found that the QIC search strategy outperformed the RCT search strategy. In the following three paragraphs, we discuss whether each of these three assumptions is likely to be justified in real-world clinical fitness landscapes.

In all of our simulations on even mildly rugged landscapes, QICs outperformed RCTs. There is evidence of some ruggedness in the real clinical fitness landscapes, although the degree of this ruggedness is unclear. Certainly, there are many known drug-drug interactions [43]; a recent study reported a 26% prevalence of clinically relevant potential drug-drug interactions between dispensed drugs to over 630,000 elderly Swedish patients [44]. Since different health care institutions routinely use different ancillary non-trial drug treatments [21], this creates the potential for treatment-by-center interactions in drug trials. There are also many other known differences in clinical practices, interventions, and health care cultures [21], [45], [46] at a variety of spatial scales (by center, by region, by nation), some of which may potentially interact with new practices or interventions being tested. As an example, it is now recognized that the effectiveness of specific patient safety practices, such as use of a checklist to prevent blood stream infections, is dependent on contextual factors including: safety culture; teamwork and leadership involvement; organizational size, complexity or financial status; financial or performance incentives or regulations regarding patient safety practices; and training and organizational incentives [33]. Recent meta-epidemiological studies found that single center RCTs showed significantly larger intervention effects than multi-center RCTs, possibly due to a variety of contextual differences between centers [47], [48], and both quantitative and qualitative treatment-by-center interactions have been reported in specific multi-center RCT results. For example, Gray [49] found substantial differences in mortality between 26 centers involved in an RCT for the treatment of lung cancer, which they postulate may be due to differences in therapy administration, and Horwitz et al. [50] observed qualitatively different responses in mortality to a treatment for heart attack in 10 of 31 centers in the RCT, which they believe may be attributed to identified differences in co-therapies at two different types of centers. As digital health care data become increasingly available, it will be interesting to apply methods developed for analyzing NK landscapes to estimate the degree of ruggedness of the actual clinical fitness landscape in different clinical domains. Such analyses may provide further guidance as to how best to search these landscapes.

There is increasing evidence that actual clinical RCTs are often statistically underpowered [51]–[54], leading to inconclusive or incorrect results. Our results are consistent with these observations, and indicate that low statistical power is a major limitation in RCT search, even in what would be considered large trials. This is due in part to the effect of diminishing returns, because larger sample sizes are needed to achieve sufficient power capable of detecting the necessarily smaller positive effect sizes available as fitness improves [55]. We show how the power of trials with even 3200 patients enrolled (considered a large trial in the real world) drops precipitously as fitness improves, meaning that to detect further improvements adequately powered RCTs would require even larger enrollments (possibly prohibitively so). Larger enrollments may require involvement from more centers and/or longer time frames that could, in turn, lead to more rugged landscapes that require even larger sample sizes for adequate power, and so on. We note that any learning strategy that does not rely on significance testing will have greater power than RCTs, so similar results are likely to be obtained under different assumptions for QIC search strategies.

We know there is large diversity in existing clinical practices and interventions between different health care institutions. However, it is not realistic to assume that practices in use are uniformly distributed among health care institutions, since there has already been extensive information sharing in the health care community through dissemination of results of RCTs (since the late 1940's), QICs (since the mid 1980's), and by less formal means for much longer. Our results show that when the median pairwise similarity of institutions is initially 90%, QIC search outperforms RCT search, even with very large trials; we have not yet tested other forms of initial clustering.

Our model assumes that all agents can conceivably move to any part of the same fitness landscape. In actuality, different health care institutions have some inherently different characteristics that are not (or not easily) changeable, such as the demographics of their patient populations; this prevents them from reaching all locations in the landscape. In future extensions to this research we plan to model this by the addition of static features with values that vary between hospitals.

In conclusion, we have performed a series of simulations using an abstract model of RCT and QIC strategies for seeking healthcare improvements. While specific quantitative results depend on the particular assumptions for the RCT and QIC strategies and landscape model, our model does capture several of the most salient characteristics of the real clinical fitness landscape and the ways in which health care institutions try to improve their positions in this landscape. The results of our simulations thus provide important insights into possible reasons for effectiveness of QICs and limitations of RCTs, and strongly support a role for collaborative learning and small, local trials in seeking improvements in the complex socio-technical environments of healthcare institutions. Our approach illustrates how modeling the evolution of medical practice as search on a clinical fitness landscape may enhance our ability to identify and understand new strategies for improving the quality and safety of medical care.
